# Biomarkers in Post-kala-azar Dermal Leishmaniasis

**DOI:** 10.3389/fcimb.2019.00228

**Published:** 2019-07-31

**Authors:** Eduard E. Zijlstra

**Affiliations:** Rotterdam Centre for Tropical Medicine, Rotterdam, Netherlands

**Keywords:** biomarkers, clinical, parasitological, biochemical, immunological, post-kala-azar dermal leishmaniasis

## Abstract

Post-kala-azar dermal leishmaniasis (PKDL) follows visceral leishmaniasis (VL, kala-azar) in 10–60% of cases. It is characterized by an asymptomatic skin rash, usually starting in the face and consisting of macules, papules, or nodules. Diagnosis is difficult in the field and is often made clinically. There is an extensive differential diagnosis, and parasitological confirmation is preferred particularly when drug treatment is considered. The response to treatment is difficult to assess as this may be slow and lesions take long to heal, thus possibly exposing patients unnecessarily to prolonged drug treatment. Biomarkers are needed; these may be parasitological (from microscopy, PCR), serological (from blood, or from the lesion), immunological (from blood, tissue), pathological (from cytology in a smear, histology in a biopsy), repeated clinical assessment (grading, photography), or combinations. In this paper, we will review evidence for currently used biomarkers and discuss promising developments.

## Introduction

**Figure 1:**
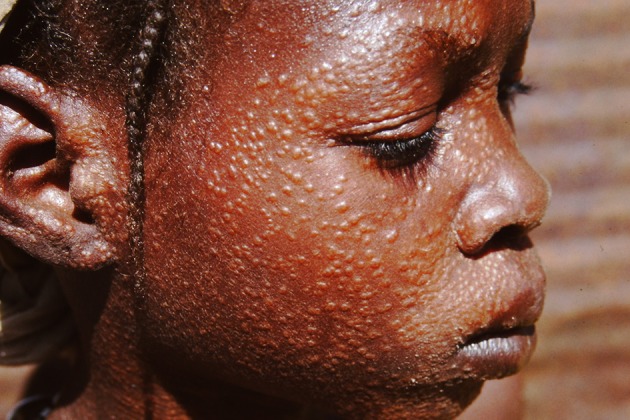
Typical papular rash in a patient from Sudan.

**Figure 2:**
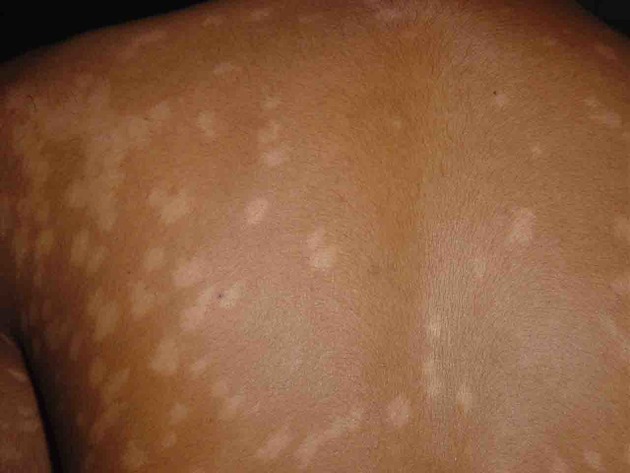
A macular rash in a patient from Bangladesh; the macules vary in size and some are confluent.

**Figure 3:**
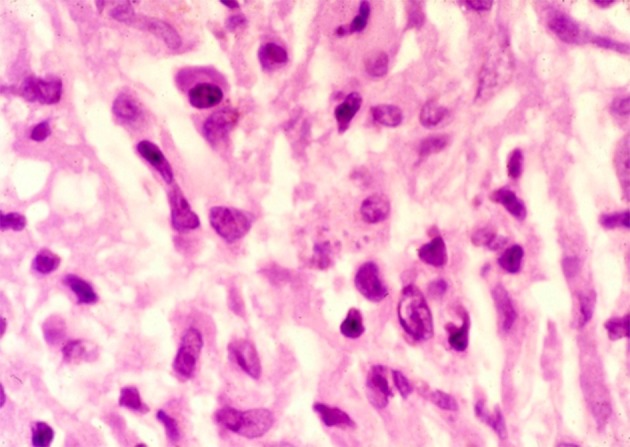
Parasites can be seen in a skin biopsy taken from a PKDL lesion.

Visceral leishmaniasis (VL, kala-azar) is most common in Asia (India, Bangladesh, Nepal), East Africa (Sudan, South Sudan, Ethiopia, Kenya, Uganda), where it is caused by *Leishmania donovani*, and South America (Brazil), where *Leishmania infantum* is the causative parasite. Interestingly, almost exclusively, VL cases caused by *L. donovani* may be followed by post-kala-azar dermal leishmaniasis (PKDL), be it not in a uniform manner. In Africa (Sudan), PKDL is much more common (up to 50–60% of VL cases) with mainly papulonodular lesions, compared with Asia (5–20%) where most cases show macular lesions (Zijlstra et al., 2003, 2017) (Figures 1, 2). In addition, the interval between VL and PKDL is short in Africa (< 12 months), whereas in Asia, it is often 3–5 years or more (Zijlstra et al., 2003). The underlying mechanism that determines development of PKDL is not completely known but may very well lie in factors that influence the evolving immune response to the parasites that can be found in the skin lesions (Figure 3). In VL patients the predominant immune response switches from a Th2 into a Th1 profile as the result of treatment, and PKDL patients are thought to have a dissociated immune response. While systemically this will be mainly Th1, the response in the skin may be still be Th2, possibly under the influence of UV light; persistence of IL-10 plays a prominent role (Gasim et al., 1998, 2000; Ismail et al., 2006; Zijlstra, 2016). The immune response varies according to clinical type and is stronger in macular PKDL than in papulonodular PKDL (Haldar et al., 1983; Saha et al., 2007; Katara et al., 2011; Mukhopadhyay et al., 2012).

Treatment induces a change in the immune response both by its antileishmanial effect and by intrinsic effects of the drugs used (Ansari et al., 2008; Mukhopadhyay et al., 2011). It follows that the effect of treatment or self-healing will be reflected in changes in clinical features, the parasite load, immune parameters in cell-mediated immunity (cell profile, cytokines, chemokines), as well as in humoral parameters such as antibody levels. In drug treatment, it is likely that parasitological cure precedes immunological cure that in its own right precedes clinical cure, with unknown intervals (Zijlstra et al., 2017). Parameters that indicate these changes may therefore be sought in each of these categories and as such have a different clinical meaning. In the case of self-healing, this process is spontaneous but most likely induced by the immune response; here, the intervals between events are equally unknown. Experience from various studies indicates that the healing process is slow, particularly in macular lesions. The reduction in size of the macular lesion is difficult to appreciate as the repigmentation process is slow; this process may take months if not years to complete (WHO, 2013; Verma et al., 2015).

Biomarkers (or biological markers) are a broad category of medical signs that reflect the medical state from outside the body and may include physical signs found on examination of the patient, basic chemical measurements, and more complex tests of blood and other tissues (Strimbu and Tavel, 2010). Identifying biomarkers in PKDL is hampered by the lack of adequate studies on diagnosis of PKDL that show considerable heterogeneity. This is due to lack of consistent reference standards, emphasizing the need for well-designed trials to assess diagnostic accuracy (Adams et al., 2013). In addition, it is essential to interpret identified biomarkers of leishmania infection in the context of the pathophysiology of PKDL, which is different from VL or cutaneous leishmaniasis (Kip et al., 2015). In recent years, considerable progress has been made in research on diagnosis, pathophysiology, and immunology of PKDL and VL, which has increased our understanding of these conditions and how they relate to each other, before and during treatment.

In practice, under field conditions, neither parasitological assessment (microscopy, PCR), biochemical parameters (antigen or antibody-based tests in blood or urine), nor immunological markers [cytokines, lymphocyte subsets, leishmanin skin test (LST)] are used and clinical assessment is often the only tool available. In hospital-based (research) laboratories, some of these tools are routinely used or under investigation.

### Special Types of PKDL

PKDL is more frequent and more severe in HIV co-infection (Abongomera et al., 2019); skin lesions may precede, accompany, or follow VL in HIV co-infection, some of which may be referred to as PKDL (Zijlstra, 2014). As the pathophysiology is completely different, this category will not be discussed.

In this paper, we will review available information on (potential) biomarkers in classical PKDL (i.e., following {successful} treatment of VL) and discuss promising developments.

## Clinical Biomarkers

### Baseline Assessment

Clinical assessment of PKDL at first presentation includes recording and description of individual lesions or groups of lesions. In addition, the presence or absence of systemic symptoms and signs is recorded; in 10% of patients, PKDL occurs concomitantly with VL, as evidenced by fever, splenomegaly, hepatomegaly or lymphadenopathy, and poor nutritional status (para-kala-azar dermal leishmaniasis) (Zijlstra et al., 2003). This distinction is important as the approach to treatment may be different: in case of suspected concomitant VL, parasitological confirmation needs to be sought and systemic treatment is given, by which the PKDL lesions are also treated simultaneously. In addition, in the case of PKDL without systemic VL, in Asia, all patients are treated, while in Africa (Sudan), only those with severe PKDL are treated as the majority of cases will self-heal (Musa et al., 2002; Zijlstra et al., 2003).

The differential diagnosis may be different in Asia and Africa but usually includes leprosy, vitiligo, and miliaria rubra (WHO, 2013). To assist field workers, guidelines for diagnosis, a PKDL atlas and an online self-teaching course have been designed, all by WHO (2012, 2013, 2017). Misdiagnosis is common (el Hassan et al., 2013) and reported in up to 26% of cases in India (Ramesh et al., 2015a). Histopathological examination of biopsies will also be of use to distinguish between differential diagnoses (el Hassan et al., 1992; Singh and Ramesh, 2013; Verma et al., 2015).

In any PKDL patient, typically the appearance of the lesions is described as macules, papules, nodules, plaques, or a mixed from. There are major differences between regions (Zijlstra et al., 2003; WHO, 2013). In Africa (mainly Sudan), a maculopapular rash (90% of cases) is most common, and in advanced cases, the papules will increase to form nodules or plaques; a pure macular rash is uncommon. In Asia, a macular rash is more common (90% of cases in Bangladesh); in hospital settings, the most common presentation may be mixed/polymorphic (53%), followed by macular lesions (23%) and papulonodular lesions (21%); unusual forms include the erythrodermic, and fibroid type, or presentations with plaques or ulcerations (Ramesh et al., 2015a; Verma et al., 2015). In contrast with Africa, advanced cases with massive lesions have been described in India (WHO, 2013; Sethuraman et al., 2017).

While the principal localization of the rash is often in the face, starting around the mouth, the rash may spread to the upper chest and arms, often corresponding with areas of the body that are not covered by clothing. Over time, or in severe cases, all parts of the body may be covered by the rash, with varying degrees of density. To describe the rash in terms of density and distribution, a grading system was designed for semi-quantitative assessment. The first was developed in Sudan and focused on distribution; later, this was refined to also include density and to describe discrepancies between these two observations. For example, a patient may have lesions all over the body (distribution grade 3), but with mostly normal skin in between (density grade 1). This would be called grade 3.1 (Table 1) (Zijlstra and el-Hassan, 2001; Musa et al., 2008).

**Table 1:** The PKDL grading system as reported from Sudan (Zijlstra et al., 2000; Zijlstra and el-Hassan, 2001).

	**Distribution**	**Density**
Grade 1	Scattered maculopapular or nodular rash on the face, with or without lesions on the upper chest or arms	Scattered lesions
Grade 2	Dense maculopapular or nodular rash covering most of the face and extending to the chest, back, upper arms and legs, with only scattered lesions on the forearms and legs	Moderate density with normal skin in between lesions
Grade 3	Dense maculopapular or nodular rash covering most of the body, including the hands and feet; the mucosa of the lips and palate may be involved; crusting and scaling may occur	No normal skin; confluent papules or nodules

**Figure 4:**
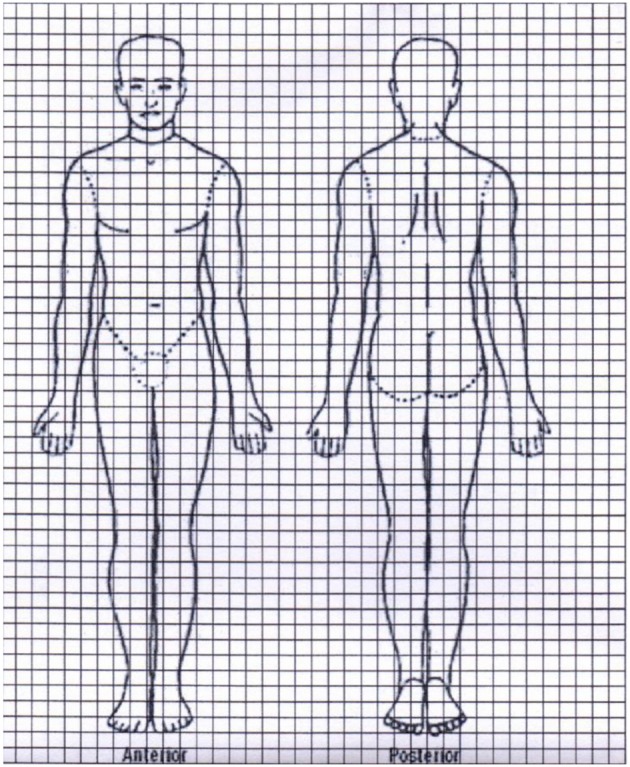
PKDL lesions are plotted on a manikin, and the number of affected squares is recorded (Mondal et al., 2016).

Alternatively, the distribution of the lesions can be plotted on a manikin as was designed in Bangladesh and the severity is assessed by counting the number of affected squares (Mondal et al., 2016) (Figure 4).

### Monitoring During Treatment or Follow-Up

While clinically, improvement or cure may be defined as flattening of lesions, improvement of dyschromia, and healing of complications, a more accurate assessment is needed particularly in drug treatment studies (Abongomera et al., 2016).

To improve accuracy of clinical (visual) description, various auxiliary methods were introduced such as clinical photography, clinical recording of severity, and distribution on various body parts, by comparing lesions as plotted on a manikin, or by comparing a clinical score in combination with a parasitological score.

Regular two-dimensional photography was introduced and standardized to accurately document lesions and allow objective comparison. This includes using the same camera, the standardized distance between camera and patient, the background of the patient, lighting in the studio, etc. The images may be interpreted by two or more independent observers who remain blinded to the patient data.

In the MSF program at Fulbaria, Bangladesh, AmBisome was used on an outpatient basis with 6 infusions of 5 mg/kg administered over 3 weeks (total dose, 30 mg/kg). After 12 months' follow-up, 34% were cured and 40% had 70–80% reduction of lesions. All nodular and papular lesions showed complete recovery while complete or significant repigmentation of macular lesions was observed in 86.5% of patients. In a subset of the latter group (*n* = 20), PCR was done on slit skin smears; all were negative. In this study, two-dimensional photography was used for documentation and comparison (Zijlstra et al., 2017; den Boer et al., 2018).

In another study in Bangladesh by MSF, with 15 mg/kg AmbiSome total dose, the lesions were also photographed by a standardized method and analyzed by three experienced physicians. In addition, a weighed core was calculated. At baseline, a percentage was assigned for the relative measure in which each affected body part (face, torso, arms, legs) contributed to the total burden of lesions.

The *improvement* of lesions was compared to the baseline for each affected body part and was recorded in percentages. The relative improvement was calculated for each body part: Relative improvement = {contribution at baseline (%) × improvement (%)}/100 (den Boer et al., 2018).

The overall improvement for each patient was calculated by: overall improvement = the sum of the relative improvement of each body part.

Final cure was defined on clinical grounds as complete resolution of nodular and papular lesions, and complete to major repigmentation of macular lesions. At 12 months, the final outcome was scored after carefully evaluating all photos of each patient in one of the following descriptive categories. This was done independently by each member of the study team (three persons) as well as by an independent external evaluator.

- Category 1: Complete resolution of nodular and papular lesions, and complete or major repigmentation of macular lesions.
- Category 2: Complete resolution of nodular and papular lesions and significant improvement of the majority of macular lesions; some macular lesions have resolved.
- Category 3: No or little improvement of lesions, but no new lesions.
- Category 4: New lesions have appeared and there is no or little improvement of other lesions.

In the study from Bangladesh by Mondal et al., a more refined method was used. Here, skin lesions were plotted by *visual* assessment in squares on a manikin (Figure 4) and the total amount of squares with lesions is counted before and after treatment. The percentage of skin lesions affected is then counted at various timepoints as a percentage: total number of squares free from lesions after treatment/total number of squares affected by the lesions at baseline (Mondal et al., 2016) (Figure 4).

In India, another method for assessment was used. In a study on the effect of miltefosine, papules and nodules were assessed rather than macular lesions and at least at three sites of the body. Two efficacy parameters were considered: a clinical score (from 0 to 6) based on numbers of papules and/or nodules, and a parasitological score (from 0–5) based on numbers of amastigotes (from 0/1000 fields to >10/field) (Sundar et al., 2013). Clinical cure was defined as follows: at 12 months, clinical score = 0 for all three locations and a parasitological score = 0 when last measured after treatment.

Three dimensional optical scanning is a novel tool to measure PKDL lesions as was demonstrated from Sudan. Optical 3-D scanning may potentially be used for surface scanning of any body part and has been tested in the assessment of burn injuries as well as mycetoma (Telfer and Woodburn, 2010; Retrouvey et al., 2018; Siddig et al., 2018). It may be a useful tool for accurate computerized measurement of lesions in patients with other tropical dermatological conditions. The 3-D scanning images may be quantified in terms of surface, circumference, and diameter, with an accuracy of 0.5 mm. In addition, the height of a lesion can be measured and sequential images can be compared to quantify changes in size and color including repigmentation as in macular lesions.

## Laboratory Biomarkers

### Parasitological Biomarkers

A confirmed diagnosis of PKDL is preferred and is mandatory in research studies.

This may be done by demonstration of leishmanial parasites by microscopy in a slit skin smear, micro-biopsy, fine needle aspirate (FNA), or conventional biopsy, with limited sensitivity of 32–50%. Parasites can more easily be found in up to 95% of mixed papulonodular lesions and in only up to 40% in macular lesions; biopsies from the buccal mucosa or the tongue have higher yield (Ramesh et al., 2015b; Verma et al., 2015).

In a recent study, comparing the slit skin smear technique (SSS) and tissue biopsy, all cases with macular lesions (*n* = 4) were negative in microscopy, while in papular lesions, 2/17 and 10/20 were positive in microscopy, in SSS and biopsy, respectively; for nodular lesions, 13/26 and 20/26 patients were microscopy positive in SSS and biopsy, respectively (Bhargava et al., 2018). The technique of performing an SSS is clearly demonstrated in Verma et al. (2013b).

PCR has higher sensitivity than microscopy, but a well-equipped lab is required. PCR has been first explored in the diagnosis of PKDL in Sudan by Verma et al. (2013b) and extensive further studies were done in Asia (Osman et al., 1998; Salotra et al., 2001; Mondal et al., 2010). Later developments included RFLP analysis and nested PCR that increased sensitivity from 69 to 93% (Schonian et al., 2003; Sreenivas et al., 2004). More recent studies show that parasite DNA is detected by PCR in a slit skin smear or biopsy in 96–100% of cases (Ramesh et al., 2015a,b; Sundar et al., 2015). PCR proved more sensitive than immunohistochemistry in biopsies from PKDL patients (Salotra et al., 2003).

qPCR or real-time PCR allows detection and quantification of a number of parasites. A summary of studies performed is given in Table 2.

**Table 2:** Summary of studies that use qPCR in diagnosis of PKDL, including those that use qPCR during follow-up.

**References**	**Method**	**Tissue obtained by**	**PKDL type**	**No tested**	**Result**
Ramesh et al., 2011	qPCR	Biopsy before treatment	Macular (2), indurated (11) polymorphic (13)	26	Range: 3–240,000 parasites/μg tissue DNA; median 667
		Biopsy after treatment		15	All negative
Ramesh et al., 2015b	qPCR	Slit aspirate			Pretreatment parasite load:
			Polymorphic	59	• In 62 who were cured: 2302 parasites/μL slit aspirate
			Macular	14	• In 11 who relapsed: 11842 parasites/μL slit aspirate
					After treatment
			All	30	• 26 negative
					• 2 <10 parasites/μL slit aspirate and were cured
					• 2 <10 parasites/μL slit aspirate and relapsed
Verma et al., 2013b	qPCR	Slit aspirate	All types	50	Range 4–70740/μL slit aspirate
			Nodular	26	Mean 9790 parasites/μL slit aspirate
			Papular/macularIn those who in slit aspirate are	24	Mean 427 parasites/μL slit aspirate
			• Microscopy positive	30	Mean 8205 parasites/μL slit aspirate
			• Microscopy negative	20	Mean 932 parasites/μL slit aspirate
			In those who in biopsy are		
			• Microscopy positive	15	Mean 15925 parasites/μL slit aspirate
			• Microscopy negative	31	Mean 791 parasites/μL slit aspirate
			1 month after treatment	17	Negative
				2	7 and 8 parasites/μL slit aspirate, 1 relapsed after 1 year
Hossain et al., 2017	qPCR Leishmania-nested PCR (Ln-PCR)	Biopsy before treatment	Macular	38	qPCR: positive 34/40; sensitivity 85% (95% CI, 70.2–94.3)
			Papular	2	Ln-PCR: positive 21/40; sensitivity 52.5 % (95% CI 36.1–68.5)
					Pretreatment parasite load by qPCR:
					• Range 1.38–4065.89 parasites/μg tissue DNA
					• Mean 295.46 parasites/μg tissue DNA
		Biopsy after treatment		40	After treatment
					• 3 remained positive in qPCR
					• 1 remained positive in Ln-PCR
Ghosh et al., 2018	qPCR	Biopsy	Macular	91	positive 83; sensitivity 91.2% (83.4–96.1%)
	Microscopy	Biopsy	Macular	91	positive 46; sensitivity 50.6% (39.9%–61.2%)
	Buffy coat	Blood	Macular	91	all negative
	qPCR	biopsy	In those who are		
			• Microscopy positive	46	IQR 9.19 (3.61–45.41)/μg tissue DNA median per μg tissue DNA^*^
					• Grade 1: 7.56 (4.5–71.22)
					• Grade 2: 8.22 (2.09–33.42)
					• Grade 3: 22.06 (3.9–43.02)
			• Microscopy negative	45	IQR 15.3 (2.99–64.7)/μg tissue DNA
	qPCR	Buffy coat	Healthy controls	86	All negative
Moulik et al., 2018	qPCR	Biopsy before treatment	All	184	Median IQR 5229 (896–50898)/μg genomic DNA
			Macular	91	Median IQR 3665 (615–21528)/μg gDNA
			Polymorphic	93	Median IQR 18620 (1266–93934)/μg gDNA
		Biopsy after treatment			
		• With miltefosine (3 m)	Macular	17	<10/μg gDNA
			Polymorphic	21	<10/μg gDNA
		• With LAMB after			
		° 3 wks	Macular Polymorphic	34 36	2128 (544–5763)/μg gDNA 2541 (650–9073)/μg gDNA
		° 6 months	All	38	5665 (1840-17067/μg gDNA
Bhargava et al., 2018	Threshold to detect parasites by		(Macular 4, papular 20, nodular 26)		
	Microscopy	SSS			4 parasites/μL SSS
	qPCR	SSS			60 parasites /μL SSS
	Microscopy	Biopsy			63 parasites/μg tissue DNA
	qPCR	Biopsy			502 parasites /μg tissue DNA

* *Number of parasites detected by microscopy: grade 1: 1–10 per 1,000 fields; grade 2: 1–10 per 100 fields; grade 3: 1–10 per 10 fields. Difference not significant (p = 0.2457)*.

There are few studies on the value of qPCR as a tool to detect parasites after treatment. Patients with a higher parasite load as measured by qPCR are at higher risk of relapse; in 30 patients studied, 26/30 were negative in qPCR 1 month after treatment, while 4 showed residual parasites, of whom 2 relapsed (Ramesh et al., 2015b) (Table 2). In a study in patients treated with miltefosine, of 15 patients sampled after 60 or 90 days post-treatment, all had become parasitologically negative as determined by qPCR from a slit skin smear (Ramesh et al., 2011). In another study, 17/19 patients became negative in qPCR 1 month after treatment with SSG or miltefosine; in 2 patients, a residual parasite load was found (7 and 8 parasites/μl slit aspirate, respectively); 1 of these relapsed (Verma et al., 2013b). Moulik et al. (2018) measured the parasite load by qPCR after treatment with miltefosine for 3 months; all had become negative by qPCR. In contrast, those treated with LAMB had higher parasite loads after 3 weeks of treatment that increased 6 months after treatment, thus predicting relapse (Moulik et al., 2018) (Table 2).

A field-friendly adaptation for DNA detection such as (closed tube) loop-mediated isothermal application (LAMP) is currently being explored (Verma et al., 2013a, 2017).

### Biochemical and Immunological Biomarkers

Antibody-based serological diagnosis with the Direct Agglutination Test (DAT) or rK39 ELISA is not helpful as antibodies may persist from the preceding VL episode. The rK39 rapid diagnostic test may be done directly on the lesions with good sensitivity but unknown specificity (Verma et al., 2013b). It has not been evaluated after treatment. A novel application is the use of the rK39 RDT in sweat samples of VL and PKDL patients with 96.6 and 94.7% sensitivity and specificity (as measured in healthy controls), respectively, with 100% concordance with blood specimens (Topno et al., 2018).

In a study from India, there was a non-significant decrease in DAT positivity rate of 75 and 66%, before and after treatment of PKDL. An assay for the migration inhibition factor showed that 70 and 100% of patients were positive, before and after treatment, respectively (Verma et al., 2015). Newer tests include the circulating immune complexes (CICs) containing glycoproteins; this test was found useful in the monitoring of PKDL patients and in distinguishing between drug-responsive and drug-unresponsive patients (Jaiswal et al., 2018).

An early study showed that IgG1 and IgG3 were significantly raised in polymorphic PKDL, while in macular PKDL, only IgG1 was elevated (Mukhopadhyay et al., 2012). The measurement of IgG1 by ELISA or by a novel rapid diagnostic test named VL sero K-SeT have recently shown to predict relapse or cure in treated VL patients. Evaluation in PKDL showed that the VL sero K-set and IgG1 ELISA supported PKDL diagnosis with a strong correlation with post VL samples, suggesting persistence of antibodies after VL. No association was found between elevated IgG1 and macular or polymorphic PKDL lesions (Marlais et al., 2018). IgG1 levels did not show a consistent decrease 1 year after PKDL treatment (Marlais et al., 2018).

Immunological parameters include measurement of immune cells (lymphocytes, monocytes, macrophages) as well as cytokines or chemokines, direct in the serum or after stimulation of peripheral blood mononuclear cells (PBMCs), or in the skin by immunohistochemistry. Serum cytokines may be measured quantitively and expressed as a ratio, referred to as the cytokine polarization index (CPI). A CPI of interferon-γ (IFN-γ) vs. IL-10 was significantly lower in PKDL cases, compared to asymptomatic VL cases, while a CPI of TNF-α vs. IL-10 was also lower, but this did not reach statistical significance (Singh et al., 2018). A similar study showed that the ratio of TNF-α (inflammatory)/IL-10 (anti-inflammatory) message was 2.66 and 1.18 in PKDL (skin biopsies) and VL (bone marrow aspirates), respectively, showing the importance of the dynamics of the cytokine profiles in various disease manifestations (Ansari et al., 2006). One study noted elevated IFN-γ transcripts after miltefosine treatment of PKDL, whereas this is not noted after antimonial therapy, suggesting that the immune response may differ according to the immunomodulatory properties of the treatment given (Ansari et al., 2008; Mukhopadhyay et al., 2011; Zijlstra, 2016). Similar effects were described for serum arginase activity (decreased) and increased serum nitrate (increased) after miltefosine treatment of PKDL, possibly indicating a macrophage activating effect (Mukhopadhyay et al., 2011).

Matrix metalloproteases (MMPs) are chemokines that are involved in tissue remodeling and leukocyte recruitment, and inhibitors thereof such as tissue inhibitor of matrix metalloproteases 1 (TIMP1) have been studied in PKDL. Using serum, MMP9 levels and the ratio of MMP9/TIMP1 were found elevated in active PKDL while patients with resolved PKDL lesions had levels similar to controls (Islam et al., 2013). This is interesting as MMP9 influences collagen degradation and mediates basement membrane modeling and TIMP inhibits activated MMP, thus possibly reflecting steps in the healing process of PKDL (Islam et al., 2013).

Adenosine deaminase (ADA) activity is an aspecific marker of the immune response and is present in all tissues (isoenzyme ADA-1) or in monocytes and macrophages (ADA-2). Serum ADA levels are raised at diagnosis of PKDL and gradually decrease during treatment. It was suggested to use the test with an rK39 RDT (specific for leishmaniasis) to increase specificity (Vijayamahantesh et al., 2016).

Cell-mediated immunity may be assessed by the *in vivo* LST. In Sudan, the LST did not discriminate between patients who, after VL, developed PKDL and those who did not as 49% and 42% of patients, respectively, had a positive LST, while all patients had been LST negative during VL (Zijlstra et al., 2000). However, patients who had parasites demonstrated in a lymph node or bone marrow aspirate during PKDL diagnosis had a positive LST in 11%, while those with a negative aspirate were significantly more likely to be LST positive (37%), suggesting a developing immune response associated with clearance of parasites (Zijlstra et al., 2000). A positive LST was associated with grade: those with PKDL grade 1 were LST positive in 39%, while those with grade 2 and 3 were LST positive in 25 and 24%, respectively (Zijlstra et al., 2000). In the only paper on the natural history of PKDL by Musa et al., 134 patients with PKDL were followed up for 12 months, using clinical assessment (grading), DAT, and LST as biomarkers. All patients had a negative LST at diagnosis. At 12 months' follow-up, those who showed self-healing had lower DAT titers over time and were more likely to develop a positive LST (89.4%), reflecting a Th1 response, whereas those with persistent PKDL after 12 months had high DAT titers and a negative LST, reflecting a Th2 response (Musa et al., 2002).

There is evidence of a stronger immunological response in macular PKDL with strong cell-mediated immunity, low numbers of parasites, and low antibody levels (only IgG1 is elevated), whereas in polymorphic PKDL, the CMI is low due to the effect of TGF-β and IL-10, with higher levels of markers for regulatory T cells, more parasites, and high antibody levels, including both IgG1 and IgG3 (markers for IL-10) (Haldar et al., 1983; Saha et al., 2007; Mukhopadhyay et al., 2012).

In another study from Sudan, the effect of immunochemotherapy in PKDL patients with persistent lesions was studied; patients were allocated standard treatment with SSG and placebo or SSG with alum/ALM+BCG vaccination. Cure was assessed by comparing grade scores before and after treatment, and by the ratio of IFN-γ/IL-10, and conversion in the LST (Musa et al., 2008). Cure rates were higher in the vaccinated group that showed increased IFN-γ production followed by conversion in the LST. The slow responders had unchanged IFN-γ and increased IL-10 levels, suggesting that IL-10 blocks the action of IFN-γ, leading to persistence of lesions; most had a non-reactive LST (Musa et al., 2008).

Other tools for cell-mediated immunity include PBMCs; increased IFN-γ may be found after *ex vivo* stimulation of blood samples with soluble leishmania antigen (SLA). In VL, these markers indicate clinical cure, but no studies have been done in PKDL (Botana et al., 2018).

## Discussion

The evolution of PKDL lesions is difficult to assess accurately, either during self-healing or after treatment. Clinical assessment as biomarker is unsatisfactory because of subjectivity and thus limited accuracy. The use of two-dimensional photograpy has limitations as it is difficult to standardize, and interpretation is subjective. Novel three-dimensional optical scanning shows promise with objective measurement of index lesions with superior accuracy and monitoring changes in size and color. Further prospective studies are awaited. Laboratory tests offer options for qualitative assessment but still have limitations, although qPCR seems the tool of choice in drug trials to assess parasite load. There is considerable heterogeneity in results from various studies, including measurements in clinical types (macular, papulonodular); this may be due to, among others, regional differences, genes targeted, clinical characteristics (duration, size, self-healing), and sampling technique. Slit skin smear or aspirate is the preferred method and is more sensitive and patient-friendly than a biopsy. Using qPCR, the parasite load can be monitored during treatment. If still positive after treatment, it is not clear what this indicates in relation to study treatment duration and whether this test will become negative over time; not all who have residual parasites as measured by qPCR will relapse. Longitudinal studies are essential in this respect. Adaptations of qPCR for use in the field are eagerly awaited.

Serological tests such as DAT, rK39 ELISA or rK39 RDT lack specificity as antibodies persist from previous VL. RDT rK39 direct on skin lesions has not been evaluated during or after treatment. Assessment of the developing immune response would be most useful as this may predict the risk of cure or relapse. *In vitro* measurement of the cytokines, chemokines, or lymphocyte subsets should be explored, and a CPI or ratio may be examined further to identify the most accurate immunological profile associated with cure. *In vivo* application of the LST deserves further study; the leishmanin needs to be well standardized and validated and requires production under good manufacturing practice.

In summary, assessment of the parasite load by qPCR in a slit skin smear or aspirate seems the most preferred biomarker to objectively measure the response to treatment and is more patient-friendly than tissue biopsy. However, a simpler and less invasive method is preferred. Given the differences of the systemic immune reponses and those of the skin, it remains to be seen if systemic biomarkers in the blood sufficiently reflect the changes in the skin.

## Conclusions

The current biomarkers in PKDL lesions are unsatisfactory and new approaches need to be explored.

For early detection of cure, a combination of a parasitological assessment using qPCR and/or an immunological assessment such as a cytokine, chemokine profile, or lymphocyte subset profile would be preferred as these are intrinsically related. Longitudinal studies are needed to describe the dynamics of this interaction in the pathophysiology of PKDL, before, during, and after cure.

## Author Contributions

The author confirms being the sole contributor of this work and has approved it for publication.

### Conflict of Interest Statement

The author declares that the research was conducted in the absence of any commercial or financial relationships that could be construed as a potential conflict of interest.
